# The dynamic regulatory network of phosphatidic acid metabolism: a spotlight on substrate cycling between phosphatidic acid and diacylglycerol

**DOI:** 10.1042/BST20231511

**Published:** 2024-10-17

**Authors:** Reika Tei

**Affiliations:** Department of Genetics, Stanford University, Stanford, CA 94305, U.S.A.

**Keywords:** cell homeostasis, lipid metabolism, lipid transfer, membranes, phosphatidic acids, phospholipases

## Abstract

Mammalian cells utilize over 1000 different lipid species to maintain cell and organelle membrane properties, control cell signaling and processes, and store energy. Lipid synthesis and metabolism are mediated by highly interconnected and spatiotemporally regulated networks of lipid-metabolizing enzymes and supported by vesicle trafficking and lipid-transfer at membrane contact sites. However, the regulatory mechanisms that achieve lipid homeostasis are largely unknown. Phosphatidic acid (PA) serves as the central hub for phospholipid biosynthesis, acting as a key intermediate in both the Kennedy pathway and the CDP-DAG pathway. Additionally, PA is a potent signaling molecule involved in various cellular processes. This dual role of PA, both as a critical intermediate in lipid biosynthesis and as a significant signaling molecule, suggests that it is tightly regulated within cells. This minireview will summarize the functional diversity of PA molecules based on their acyl tail structures and subcellular localization, highlighting recent tools and findings that shed light on how the physical, chemical, and spatial properties of PA species contribute to their differential metabolic fates and functions. Dysfunctional effects of altered PA metabolism as well as the strategies cells employ to maintain PA regulation and homeostasis will also be discussed. Furthermore, this review will explore the differential regulation of PA metabolism across distinct subcellular membranes. Our recent proximity labeling studies highlight the possibility that substrate cycling between PA and DAG may be location-dependent and have functional significance in cell signaling and lipid homeostasis.

## Introduction

Phospholipids are major building blocks for cell membranes as well as important participants in cell signaling and regulation. Mammalian cells utilize over 1000 species of lipids to manage diverse and dynamic physiological functions and responses [1,2]. Membrane lipids regulate protein functions and localizations, either directly via lipid–protein binding or indirectly by changes in physical and chemical membrane microenvironment. In turn, proteins such as lipid-metabolizing enzymes and lipid-transfer proteins control metabolism and trafficking of membrane lipids, resulting in complex networks of lipids and proteins [3,4]. In the past century, studies have revealed dozens of lipid-metabolizing enzymes and their functions, including their highly interconnected network of phospholipid biosynthesis [5,6]. However, mechanisms of these functions — how they are regulated in orchestra to have the right lipid, at the right place, at the right moment — are largely unknown. Mammalian cells likely achieve lipid homeostasis by series of cross-regulations and feedback loops [7], and failure in this coordination can lead to pathological outcomes [8]. Moreover, cells seem to have various priorities for each lipid homeostasis, i.e. certain lipids are under tighter control than others.

For example, phosphatidylcholine (PC), the most abundant lipid that accounts for 40–60% of cellular lipidome, is one of the lipids that are under strict regulation, and their levels are highly resilient against various disturbance [7]. Previous studies showed that facilitating PC synthesis via genetic manipulation failed to increase total PC levels because cells adapted these changes by concomitantly increasing degradation of PC [9–11], and likewise, reduced rate of PC synthesis was compensated by decreased PC turnover [12]. Similar homeostatic regulations have been observed for phosphatidylethanolamine (PE) and phosphatidylserine (PS), where cells managed to maintain their levels constant despite exogenous manipulations [7,13]. How do cells sense such disturbance and tune phospholipid biosynthesis and metabolism to promptly achieve homeostatic adaptation? It is a truly fascinating question in cell biology as well as pharmacology, as its understanding potentially leads to development of new clinical approaches to pathological malfunctions in lipid homeostasis.

**Figure 1.**
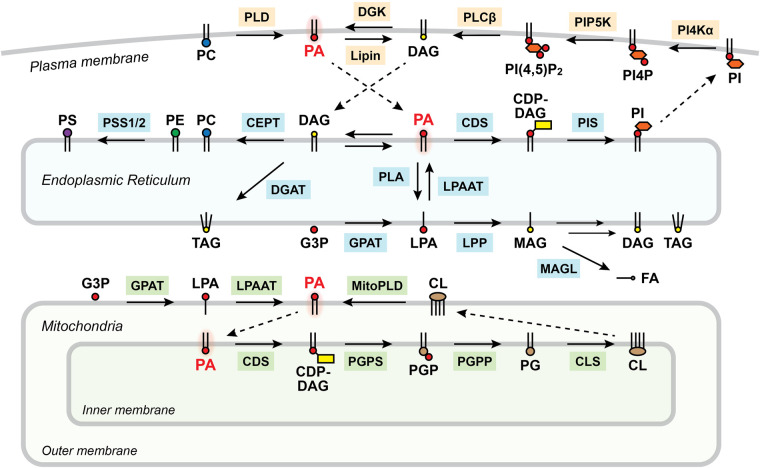
Phosphatidic acid (PA) is a major hub for phospholipid synthesis and metabolism pathways. Scheme showing major pathways of PA synthesis and metabolism in mammalian cells. Abbreviations for lipids: CDP-DAG, cytidine diphosphate diacylglycerol; DAG, diacylglycerol; FA, fatty acid; G3P, glycerol 3-phosphate; LPA, lysophosphatidic acid; MAG, monoacylglycerol; PA, phosphatidic acid; PC, phosphatidylcholine; PE, phosphatidylethanolamine; PG, phosphatidylglycerol; PGP, phosphatidylglycerophosphate; PI, phosphatidylinositol; PI4P, phosphatidylinositol 4-phosphate; PI(4,5)P_2_, phosphatidylinositol 4,5-biphosphate; TAG, triacylglycerol. Abbreviations for enzymes: CDS, cytidine diphosphate diacylglycerol synthase; CEPT, choline/ethanolamine phosphotransferase; CLS, cardiolipin synthase; DGAT, diacylglycerol acyltransferase; DGK, diacylglycerol kinase; LPAAT, lysophosphatidic acid acyltransferase; LPP, lipid phosphate phosphatase; MAGL, monoacylglycerol lipase; PGPP, phosphatidylglycerophosphate phosphatase; PGPS, phosphatidylglycerophosphate synthase; PI4Kα, phosphatidylinositol 4-kinase alpha; PIP5K, phosphatidylinositol 4-phosphate 5-kinase; PIS, phosphatidylinositol synthase; PLA, phospholipase A; PLD, phospholipase D.

Phosphatidic acid (PA) is one of the most important lipids for phospholipid homeostasis. Despite its abundance being 1% or less of total lipids, it has a pivotal role as a key intermediate for biosynthesis of most of the other phospholipids [14,15]. Figure 1 shows an overview of PA synthesis and metabolism pathways. PA is mainly synthesized on the plasma membrane via phospholipase D (PLD) pathway [16,17] or phospholipase C (PLC)/diacylglycerol kinase (DGK) pathway [18,19]. Additionally, PA can be synthesized on other subcellular locations such as endoplasmic reticulum (ER) and mitochondria via lysophosphatidic acid acyl transferase (LPAAT) pathway [20], mitoPLD pathway [21], and NAPE-PLD pathway [22]. For PA metabolism, there are three main pathways, each has distinct contribution to phospholipid biosynthesis: PA–DAG route fuels synthesis of PC and PE (which can further be converted to PS) via Kennedy pathway [23,24], PA–cytidine diphosphate diacylglycerol (CDP-DAG) pathway leads to synthesis of phosphatidylinositol (PI) and phosphatidylglycerol (PG) [25], and PA–lysophosphatidic acid (LPA) conversion can further lead to degradation into fatty acids, redirecting resources to *de novo* lipid synthesis [26–28]. In addition to these direct and indirect roles PA plays in maintaining lipid homeostasis, PA itself is also an important signaling molecule. PA is involved in various cellular processes including cell survival [29], cytoskeleton remodeling [30,31], receptor endocytosis [32,33], and membrane trafficking [34–37], mediated through its interaction with PA-binding proteins [38,39]. These pleotropic functions of PA, both as a key intermediate in lipid biosynthesis and as a potent signaling molecule, suggest that PA must be under tight control in mammalian cells. This minireview will analyze the functional diversity of PA molecules and discuss the strategies cells employ to regulate and maintain PA levels, based on the latest findings and insights. Especially, recent studies suggest that the substrate cycling between PA and DAG, where each serves as a reaction substrate for the other, may play crucial roles in cell signaling and lipid homeostasis. I will introduce the potential underlying mechanisms of location-dependent regulation of PA/DAG substrate cycling, possibly contributing to local signal prolongation and propagation.

## Main

### Functional variety of different PA species

Cells produce a number of phospholipid species with varying length and degree of saturation of the fatty acid chains [2]. While phospholipids are classified by their head groups, their acyl tails play significant roles in their functions. Saturated acyl chains, which lack any double bond, generally have less entropy and favor tight packing within lipid bilayer. On the other hand, unsaturated acyl tail species can introduce higher flexibility and fluidity to the membrane. As such, each lipid behavior, such as submembrane localization and accessibility to membrane-associated proteins, is influenced by physical properties of their acyl chains. Chemical properties of acyl tails also contribute to functional diversity of each lipid. For example, unsaturated lipids often show distinct metabolic pathways from saturated ones, because their double bonds can undergo oxidation [40]. Moreover, the length and saturation of fatty acid chains determine their overall shapes, which can make some of them ‘a better fit’ for each lipid-binding protein. Collectively, phospholipid species with different acyl chains, with their physically and chemically distinct properties, can exert diverse functions in membrane dynamics and cell signaling.

The most common PA species found in mammalian cells contain 16:0 (palmitoyl), 18:0 (stearoyl), 18:1 (oleoyl), or 18:2 (linoleoyl) fatty acid at the sn-1 and sn-2 positions of the glycerol backbone [41]. The main source that contributes to PA acyl chain composition is the substrate preference of PA-producing enzymes. For example, phospholipase Ds (PLDs) hydrolyzes PC to produce PA predominantly in mono-unsaturated species, such as PA 16:0/16:1 and PA 16:0/18:1 [42]. On the other hand, diacylglycerol kinases (DGKs), which convert diacylglycerol (DAG) to PA, have 10 isoforms with various acyl chain preferences, contributing to the diverse profile of PA species [39]. Interestingly, studies revealed that some PA-binding proteins exhibit selectivity to specific PA species [39]. For example, Praja-1 and Synaptojanin-1 are found to bind preferentially to minor PA species 18:0/22:6, rather than to the abundant ones such as PA 16:0/18:1 [43,44]. In another study using PA analogs with photoconvertible azobenzene moiety in one or both of their fatty acid chains, we found that these photoswitchable PA analogs can be incorporated into live cell membrane and mimic the known biological functions of PA, but they show distinct bioactivity and metabolism under light-adapted (*cis*, ‘bent’) vs. dark-adapted (*trans*, ‘straight’) forms [45]. The PA analog with both of their acyl tails in the ‘bent’ form exerted much higher activity in positive regulation of mTOR signaling and negative regulation of Hippo signaling [45]. This specific form was also most metabolically active, being converted into azobenzene-containing lipid derivatives corresponding to DAG, fatty acid, and PC. Because the ‘bent’ acyl tail is structurally more akin to unsaturated fatty acids, it would align with the notion that kinks in the acyl chains can provide higher membrane fluidity and accessibility to effector proteins.

**Figure 2.**
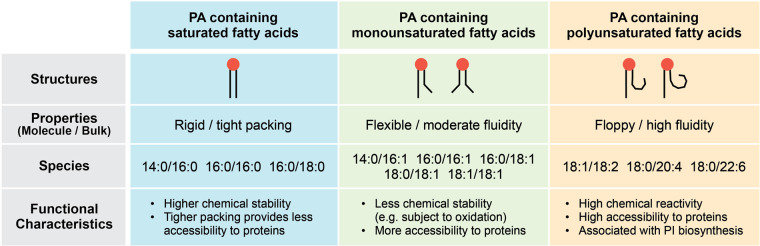
Phosphatidic acid (PA) species with distinct acyl tails and their properties. A summary table of three categories of PA species, each containing saturated, monounsaturated, or polyunsaturated fatty acids, describing their structural and molecular properties, species found in mammalian cells, and key functional characteristics.

PA shares an overall similar acyl chain profile as PC and DAG, both of which are most enriched in 16:0/18:1 species [46], reflecting their high metabolic connectivity. This is distinct from PI, which is most enriched on 18:0/20:4 [47]. Interestingly, under phospholipase C (PLC)-stimulated conditions where PI(4,5)P_2_ is converted to DAG, a concomitant increase in PA was observed on species highly enriched in poly-unsaturated fatty acids such as PA 18:0/20:4, reflecting a characteristic acyl chain composition of PI [46]. Moreover, these PA species were also preferentially resynthesized back into PI, likely via PA–CDP-DAG–PI pathway [46]. Because PLC-mediated production of PA takes place on plasma membrane, it seems critical for these PA molecules to get trafficked to ER, where they can meet PI-synthesizing enzymes. The properties of various PA species discussed above are summarized in Figure 2.

### Spatiotemporally defined PA pools and their distinct functions

The production of PA on the inner leaflet of plasma membrane has been observed at various conditions, including stimulation of PLC or PLD [18,48,49]. While it makes plasma membrane the most pronounced location of PA synthesis, PA pools on ER are undoubtedly important for lipid metabolism, because of ER being a major hub for lipid biosynthesis [50,51]. Moreover, PA functions have been implicated on other subcellular locations, including mitochondria [52], Golgi [53], endosomes [54], and nuclei [55], among many others where the locations responsible are yet to be identified [14,29,38,39]. Studying locational dependency of PA functions has been challenging, because exogenous PA supplementation has no control over its destination, and genetic manipulation of PA-producing enzymes have unclear outcomes due to their not fully understood regulatory mechanism of their activity and localization. To decouple the functional diversity of spatially defined PA pools, we recently developed optogenetic-based tools to control the location of PA synthesis in living cells [42,56]. These tools are termed optoPLD [42], or superPLD [56] for its directed evolution mutant. They harness an engineered bacterial PLD, whose location is controlled using a light-induced dimerization system [42] and activity is optimized for mammalian intracellular environment by directed evolution [56]. SuperPLD is ∼30–100 times more active than optoPLD in mammalian cells and capable of hydrolyzing 2–4% of total PC in 30 min [56]. By using optoPLD, we revealed that PA made on plasma membrane, but not on other organelle membranes such as ER, Golgi, and endosomes, can exert negative regulation of Hippo signaling [42]. Lazcano et al. [57] also demonstrated that PA produced on plasma membrane by optoPLD effectively triggers the nuclear translocation of a kinase IP6K1, which repress expression of myo-inositol-3-P synthase that mediates inositol synthesis.

**Figure 3.**
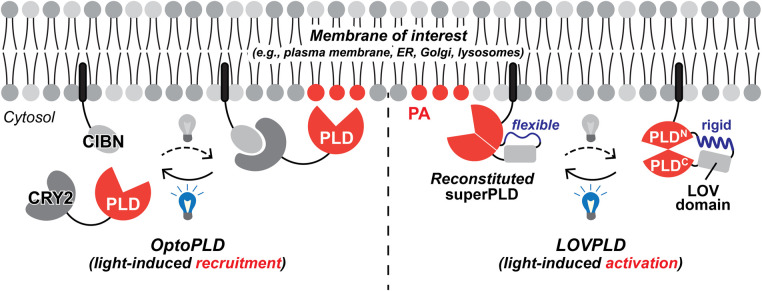
Light-activated phospholipase Ds (PLDs) for spatiotemporal control of PA production. Left design depicts optoPLD, for light-induced recruitment of PLD_PMF_ (derived from Streptomyces. PMF) or superPLD (a directed evolution mutant of PLD_PMF_) [56]. Right design depicts LOVPLD, which enables light-induced activation of superPLD [58]. In optoPLD, blue light induces dimerization of CRY2 and CIBN, mediating the recruitment of PLD_PMF_ or superPLD to the membrane of interest to which CIBN is tagged. In LOVPLD, a light-oxygen-voltage-sensing (LOV) domain is inserted into superPLD as a clamp that distorts its original structure until the blue light relaxes the LOV domain structure, allowing superPLD to reconstitute. PLD^N^ and PLD^C^ indicate N- and C-terminal halves of superPLD, respectively.

While decoupling the effects of spatially defined PA pools sheds light on the mechanisms of their functions, the metabolic pleiotropy of PA as a major lipid synthesis hub complicates the landscape. During genetic and pharmacological manipulation of PA as well as many other lipids, it is often not clear if the lipid *per se* or its metabolite is the role in action because of their intricate and intertwined metabolic networks. To address this, a fine temporal control of lipid production is needed. To control production of PA at the right place *AND* at the right time, we further harnessed protein engineering to introduce a light-mediated allosteric control within the superPLD structure. This was done by inserting a light-responsive photoswitch LOV domain into one of the flexible loops of superPLD, such that superPLD remains in inactive form in dark until light-induced relaxation of LOV domain allows superPLD to reconstitute its structure and restore its activity [58]. This light-switchable superPLD, termed LOVPLD, is a clear contrast from the original superPLD, where light only mediates the recruitment of PLD enzyme and thus the unrecruited enzyme casts certain background activity (Figure 3). Thus, LOVPLD greatly enhances our capability to decouple the spatial and temporal functional diversity of PA in live cells.

### Physiological importance of PA homeostasis

Lipid homeostasis is the fundamental principle of lipid synthesis and metabolism. Cells somehow make sure that the right species of lipids exist at the right place and only at the right moment, by coordination of dozens of lipid-metabolizing enzymes, lipid-transfer proteins, and other biomolecules. Cells with disturbed lipid metabolism are likely ill-fated. For example, PS overproduction is found in Lenz-Majewski hyperostotic dwarfism disease, caused by mutated PS-synthesizing enzyme PSS1 that lack product inhibition [59,60]. Altered lipid metabolism is also common trait in cancer cells, who promote synthesis of many lipids including PA, DAG, and PC, to support their rapid growth and proliferation [61,62]. Interestingly, severe functional outcomes of altered PA metabolism are often found in the context of PA abundance rather than depletion. For example, lipin deficiency and consequent PA accumulation are often linked to pathology [63]. Lipin is a PA-metabolizing enzyme that degrades PA into DAG [64], whose reaction counteracts with PA synthesis from DAG by DGK. Loss-of-function mutation in Lipin-2 causes Majeed's syndrome, a rare autosomal recessive autoinflammatory disorder [65]. Knocking out two of three lipin isoforms, Lipin-1 and Lipin-2, results in embryonic lethality in mice [66]. This is a stark contrast from the fact that the mice deficient in both PLD isoforms, PLD1 and PLD2, show no apparent defect in their health [67]. The latter aligns with our observation that PLD1/2 double-knockout HEK cells showed no decrease in cellular PA levels [42]. The apparent lack of functional outcome in PLD knockout highlights the redundancy in PA-synthesizing pathways, where loss of one pathway can be compensated by others. Moreover, blocking PA synthesis via DGK, which phosphorylates DAG to PA, triggers PLD activation to replenish PA, potentially via DAG-mediated activation of protein kinase C (PKC). Thus, DAG–PKC–PLD–PA pathway stands out as a feedback mechanism that cells harness to keep constant supply of PA. However, little is known about other regulatory networks for PA homeostasis, including how cells utilize so many different pathways of PA synthesis and metabolism to maintain PA levels.

### Regulation of PA metabolism and homeostasis

**Figure 4.**
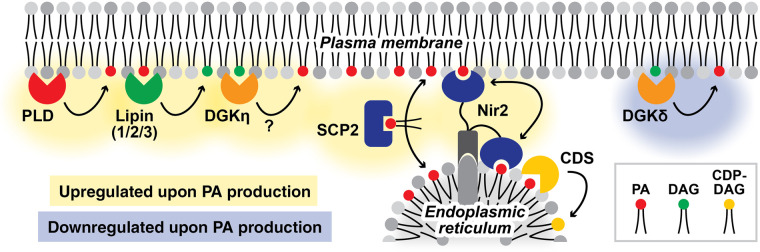
Key players in metabolism and trafficking of PA produced on plasma membrane, revealed by Feeding–Fishing proteomics. CDP-DAG, cytidine diphosphate diacylglycerol; CDS, CDP-DAG synthase; DAG, diacylglycerol; DGK, diacylglycerol kinase; PA, phosphatidic acid; PLD, phospholipase D; SCP2, sterol carrier protein 2. Nir2 is also known as PITPNM1.

Previously, we observed that manipulative increase of PA level using superPLD led to concomitant increase in PG [58,68], indicating that cells route excess PA molecules into CDP-DAG pathway. At the same time, we did not observe any decrease in PC, a substrate for superPLD, so certain amount of PA is likely used to replenish PC via Kennedy pathway. Interestingly, PA levels showed higher fold enrichment when PA was made on plasma membrane rather than on other membranes such as lysosomes [58,68], suggesting slower turnover of PA on plasma membrane. To understand the regulatory mechanisms of this differential PA metabolism, we combined superPLD-based membrane editing and proximity labeling to perform ‘Feeding–Fishing’ proteomics. This was done by using superPLD to ‘feed’ PA molecules onto membrane of interest and proximity labeling enzyme TurboID to ‘fish’ out proteins associated with the same membrane. Feeding–Fishing proteomics were conducted on two distinct membranes where endogenous PLDs are reported to localize — plasma membrane and lysosomes [16] — and we mapped the proteins that are enriched or depleted upon PA feeding (key hits summarized in Figure 4). Among the proteins that showed increase on PA-fed membranes were lipins, which degrade PA to DAG, and several lipid-transfer proteins including Nir2, SCP2, PDZD8, TEX2, and specific members of VPS13 family proteins (VPS13B for PA-fed plasma membrane and VPS13C for PA-fed lysosomes) [68]. Interestingly, all lipin isoforms (Lipin-1/2/3) were found enriched on PA-fed plasma membrane, while only Lipin-1 was detected on lysosomes at much lower abundance [68]. As for the proteins that showed decreases on PA-fed membranes, a much fewer number of lipid-metabolizing enzymes and lipid-transfer proteins were identified, with the most noticeable one was DGKδ, which synthesizes PA from DAG. These findings overall indicate that cells attempt to counteract excess PA levels by boosting PA degradation by lipins, transferring PA molecules to other membranes (e.g. to ER, where they are converted to CDP-DAG by CDS1/2), and tuning down PA synthesis by DGK. However, the higher enrichment of lipins on plasma membrane is counter-intuitive to the lipidomics analysis results showing slower PA turnover on this membrane.

These apparently contradictory findings point to a new possibility that PA/DAG substrate cycling may act as a reservoir to keep both molecules from being degraded via other pathways. Substrate cycling, or futile cycle, is a system where two opposing biochemical reactions run simultaneously [69]. Well-known examples of this include interconversion of glucose/glucose-6-phosphate and fructose-6-phosphate/fructose-1,6-biphosphate, both of which occur in glycolysis and gluconeogenesis. Such a pathway is generally regarded as energy-wasting ‘futile’ cycle, but recently, their beneficial functions and utilities have been increasingly appreciated in certain cases [69]. When it comes to PA and DAG, as both molecules are subject to degradation and routing to other phospholipid synthesis (via CDP-DAG pathway and Kennedy pathway, respectively), facilitating their interconversion seems to be a reasonable strategy for cells when their roles in signal transduction as second messengers are needed. Considering that plasma membrane being a hot spot for cell signaling, it makes sense that cells activate PA/DAG substrate cycling on this membrane by utilizing lipid-metabolizing enzymes that catalyze PA/DAG interconversion. Potentially aligning this hypothesis, one of the DGK isoforms, DGKη, was found to be enriched upon PA feeding, but only on plasma membrane and not lysosomes [68]. The notion that PA/DAG substrate cycling contributes to signal amplification might explain why PA produced on plasma membrane seems to be consistently more functionally effective than PA made on other membranes, such as ER [42,57,58]. Another observation relevant to this theory was recently made by Tamas Balla's group [46]. In their study tracking the metabolic fate of *de novo* synthesized PA using radiolabeled substrate, they confirmed PI synthesis via PA–CDP-DAG–PI pathway. Interestingly, the PI formation showed a marked decrease (∼50%) when DGK inhibitor was added to the cells, indicating that at least some of the PI must have been generated from PA molecules that have gone through PA/DAG cycling [46]. Collectively, these recent studies support the emerging concept that PA/DAG substrate cycling may be important for cell signaling and lipid homeostasis, specifically maintaining levels of PA as well as other lipids such as PI.

## Conclusion and outlook

PA is the central hub for phospholipid biosynthesis as well as the potent signaling molecule. Altered PA metabolism and disordered homeostasis lead to malfunctional outcomes, and cells maintain levels of each PA species with spatiotemporal precisions. Such differential and specific regulation is likely achieved by a coordination of several factors, including the acyl chain preference and localization of lipid-modifying enzymes and lipid-transfer proteins, as well as bulk physical properties of each lipid species that determine their behavior in membranes. The species of PA as well as their localization matter for their fate and functions. Emerging studies using tools for spatially controlled PA production have revealed that PA produced on different membranes, such as plasma membrane vs. ER, have differential effects on their roles in cell signaling and lipid metabolism. I envision that our most recent development of the light-activable membrane editor LOVPLD [58] would be employed to generate spatially defined PA pools in live cells in the system to study the PA functions whose spatial association is yet to be determined.

It is truly fascinating to think how proteins sense the head group and acyl chain of each lipid to assign them to designated metabolic fates and localizations. In this minireview, I highlighted recent findings that shed light on the regulatory mechanisms of PA metabolism and homeostasis. Particularly, the concept of substrate cycling between PA and DAG, their potential functional significance in cell signaling and lipid homeostasis, and the suggested mechanism underlying its location-dependent regulation are introduced. I hope that the insights shared here contribute to a better understanding of lipid biology, whose field is constantly evolving through the development and application of molecular tools and multi-omics technologies, enabling researchers to manipulate membrane lipids, analyze their functional effects, and identify the underlying molecular basis associated with them. Ultimately, continued research will pave the way for a comprehensive decoding of the functional diversity of lipids.

**Perspective**

- PA is the central hub of phospholipid biosynthesis, and its level is spatiotemporally controlled in mammalian cells to maintain homeostasis.
- Substrate cycling between PA and DAG may play a significant role in regulating cell signaling and lipid metabolism, because their interconversion protects these signaling lipids from being consumed for other phospholipid biosynthesis.
- Continuous development and applications of new tools, such as the light-activable superPLD (LOVPLD) to generate spatially defined PA pools on the membrane of interest in live cells, would help decode the diverse and pleiotropic functions of lipids.

## Abbreviations

DAG: diacylglycerol
DAGK: diacylglycerol kinase
ER: endoplasmic reticulum
PA: phosphatidic acid
PC: phosphatidylcholine
PE: phosphatidylethanolamine
PG: phosphatidylglycerol
